# Pulmonary *Madurella mycetomatis* mycetoma secondary to knee eumycetoma, Senegal

**DOI:** 10.1371/journal.pntd.0009238

**Published:** 2021-03-25

**Authors:** Khadim Diongue, Mamadou Alpha Diallo, Lamine Sarr, Mame Cheikh Seck, Ludivine Bréchard, Mouhamadou Ndiaye, Aida Sadikh Badiane, Stéphane Ranque, Daouda Ndiaye

**Affiliations:** 1 Laboratory of Parasitology and Mycology, Aristide Le Dantec University Hospital, Dakar, Senegal; 2 Service of Parasitology-Mycology, Faculty of Medicine, Pharmacy and Odontology, Cheikh Anta Diop University of Dakar, Dakar, Senegal; 3 Service of Orthopedics, Aristide Le Dantec University Hospital, Dakar, Senegal; 4 University Hospital Institute Méditerranée Infection, Marseille, France; Universidade Federal do Para, BRAZIL

## Abstract

Mycetoma is a neglected tropical disease which is endemic in Senegal. Although this subcutaneous mycosis is most commonly found on the foot, extrapodal localisations have also been found, including on the leg, knee, thigh, hand, and arm. To our knowledge, no case of blood-spread eumycetoma has been reported in Senegal. Here, we report a case of pulmonary mycetoma secondary to a *Madurella mycetomatis* knee eumycetoma. The patient was a 41-year-old farmer living in Louga, Senegal, where the Sudano-Sahelian climate is characterised by a short and unstable rainy season and a steppe vegetation. He suffered a trauma to the right more than 20 years previously and had received treatment for more than 10 years with traditional medicine. He consulted at Le Dantec University Hospital in Dakar for treatment of a right knee mycetoma which had been diagnosed more than 10 years ago. He had experienced a chronic cough for more than a year; tuberculosis documentation was negative. Grains collected from the knee and the sputum isolated *M*. *mycetomatis*, confirmed by the rRNA gene ITS regions nucleotide sequence analysis. An amputation above the knee was performed, and antibacterial and antifungal therapy was started with amoxicillin-clavulanic acid and terbinafine. The patient died within a month of his discharge from hospital.

## Case presentation

**Fig 1:**
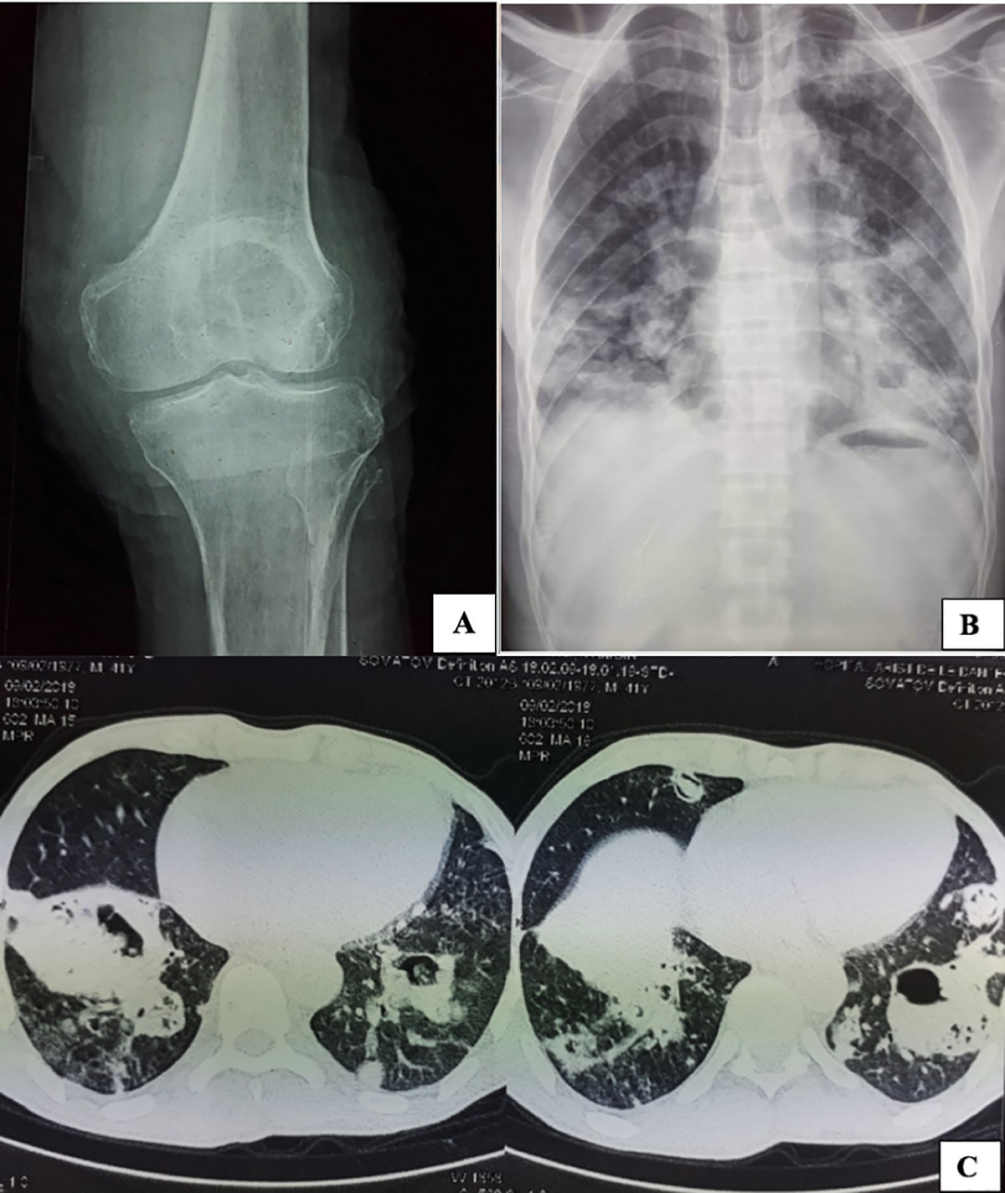
(**A**) Frontal knee X-ray showing osteolysis at the kneecap and a significant bone demineralisation induced by lack of support on the limb. (**B**) Chest X-ray showing predominant pulmonary opacities at the lower lobes with a cave in the right lower lobe. (**C**) CT-scan showing bilateral nodules and consolidations with cavities, some of which contained fungus balls located in the lower lobes.

A 41-year-old male farmer consulted at Le Dantec University Hospital in Dakar, Senegal for treatment of a right knee mycetoma which had been diagnosed more than 10 years previously at the regional hospital of Louga, Senegal. He was living in Louga, an area located 203 km northeast of Dakar, Senegal, with a Sudano-Sahelian climate, a short and unstable rainy season and steppe vegetation. He had experienced a knee trauma more than 20 years previously that had been treated for more than 10 years by a traditional practitioner with plant (leaves and bark) infusions. Upon admission, he was thin and in poor general condition, with pale mucous membranes and a body mass index of 17. He was limping and his knee mobility was limited, with 80° flexion. The X-ray showed a very aggressive infectious process involving the kneecap and surrounding areas **(Fig 1A)**. Routine biological investigations (haematology and biochemistry) were within the normal range, with the exception of an elevated C-reactive protein at 287 mg/L. He was admitted to the surgical department for amputation. Surgical treatment was, however, delayed due to a chronic cough that had been evolving for more than a year. The various analyses performed to investigate tuberculosis infection, including a tuberculin skin test, bacilloscopy, and GeneXpert MTB/RIF, were negative. An amputation above the knee, an indication that had been debated by the orthopaedic surgical staff, was performed at the patient’s request. Grains collected during the surgery were sent to the Parasitology and Mycology laboratory for mycological analysis.

The grains (visible to the naked eye) were dark brown to black and were small in size, 0.5 to 1 mm, round, sometimes entangled, and were hard with a few soft specimens. The grains were first washed to remove impurities by placing them in a test tube containing sterile physiological saline. The contents were mixed and then centrifuged at 3,000 rpm for 2 minutes. This procedure was repeated twice with the sediment remaining at the bottom of the tube. The remaining sediment was viewed in a Petri dish with a little sterile physiological saline. Clean grains were taken with single-use forceps for further analysis. Direct microscopic examination of a 20% potassium hydroxide mount of clean grains, after crushing between slide and coverslip, revealed septate fungal hyphae with expanded terminal cells. The washed grains were inoculated on Sabouraud dextrose agar plates with chloramphenicol, either supplemented or not supplemented with cycloheximide, and incubated at both 25°C and 37°C in ambient conditions.

After surgery, the patient continued to complain of a cough, and a chest X-ray was requested. It revealed predominant pulmonary opacities at the lower lobes with a cave in the right lower lobe **(Fig 1B)**. The chest CT-scan revealed bilateral nodules and consolidations with cavities, some of which contained fungus balls located in the lower lobes **(Fig 1C)**.

**Fig 2:**
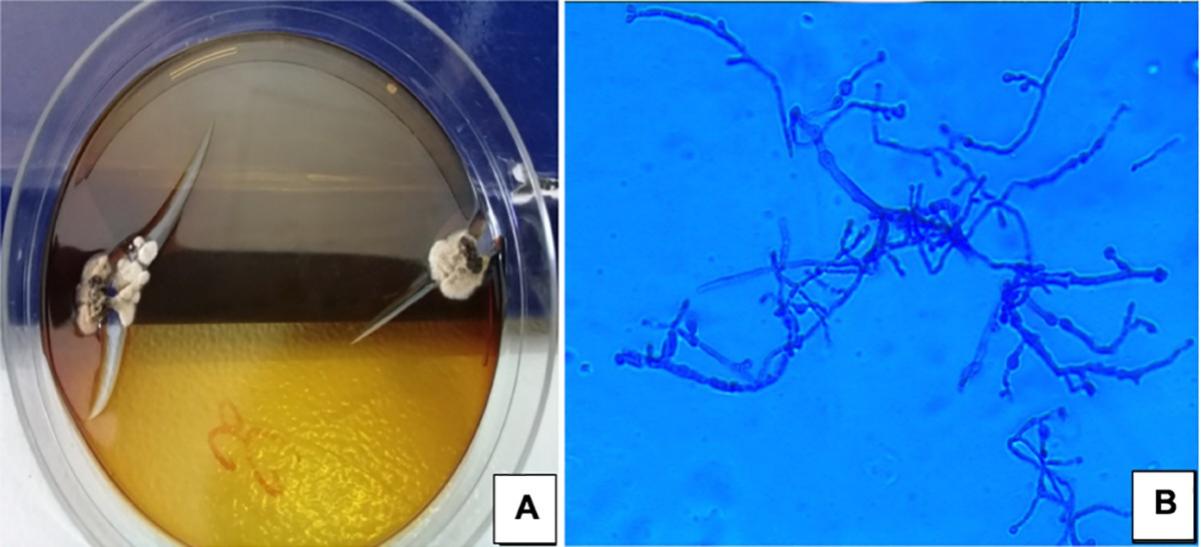
Culture on Sabouraud dextrose agar showing flat and dry colonies adhering to the agar, white to yellowish brown in colour, slightly folded with a brown diffusible pigment (A) and microscopic features of the colonies showing a sterile mycelium composed of septate hyphae with several chlamydospores (B).

At the same time, sputum seeded with black grains were sent to the laboratory and was treated as described for samples from the knee. After several days of incubation, flat colonies adhering to the agar appeared from the knee grain culture. They were white to yellowish brown, becoming brownish, slightly folded with a brown diffusible pigment. Under the microscope, a sterile mycelium composed of septate hyphae with several chlamydospores was observed **(Fig 2)**.

**Fig 3:**
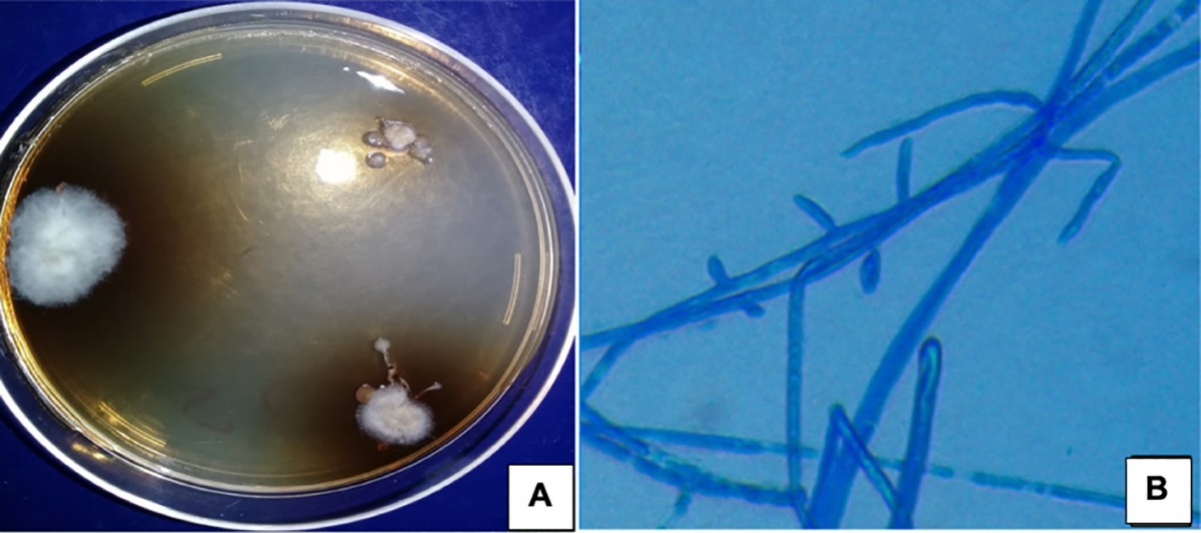
Cultures on Sabouraud dextrose agar showing dry colonies, white with a greyish surface mycelium and a diffusible brown pigment (A). The microscopic features of the colonies displayed septate hyphae with branched conidiophores bearing pyriform conidia (×1000).

In contrast, culture of the grains contained in the sputum were contaminated by colonies of *Candida albicans* after 2 attempts. The grain washing protocol was therefore modified. The physiological saline solution was supplemented with a few drops of 10% povidone iodine. This led to the isolation of dry colonies which were white with a greyish surface mycelium and a diffusible brown pigment. Microscopically, septate hyphae with branched conidiophores bearing pyriform conidia were observed **(Fig 3)**.

In both cases, the species were identified as *Madurella mycetomatis*. These identifications were confirmed by analysing the nucleotide sequence of the ITS regions of the rRNA gene, respectively from the grains and the colonies, using ITS1 (TCCGTAGGTGAACCTGCGG) and ITS4 (TCCTCCGCTTATTGATATGC) primers. DNA extraction was performed using the EZ1 Advanced XL instrument (Qiagen Hilden, Germany). Grains or a portion of colonies on culture medium were introduced into a bead tube containing 700 μl of lysis buffer G2 (supplied with the EZ1 DNA Investigator kit, Qiagen). This was followed by 2 runs of mechanical lysis with FastPrep-24 and by centrifugation at 10,000 rpm for 1 minute. Finally, 200 μl of the supernatant was removed and placed in an EZ1 flat tube before launching the EZ1 Advanced XL instrument according to the manufacturer’s instructions. Genomic DNA was extracted and eluted in 100 μl elution buffer. PCR reactions were performed in a final volume of 50 μL, containing 25 μL of AmpliTaq Gold 360 master mix, 1.5 μL of both forward and reverse primers (20 pmol), 5 μL template DNA, and 17 μL RNase free water. The PCR program was as follows: 94°C for 10 minutes, followed by 40 cycles at 94°C for 20 seconds, 53°C for 30 seconds, and 72°C for 1 minute, with a delay at 72°C for 7 minutes. After revelation in 2% agarose gel stained with ethidium bromide visualised under an UV illumination (E-gel Imager, Thermo Fisher Scientific, Massachusetts, United States of America), the amplicon was purified using the Sephadex G50 DNA purification kit. Sequencing reactions were processed using a 3500 Genetic Analyzer (Applied Biosystems, Massachusetts, USA), and the sequences obtained were assembled using the CodonCode Aligner software (CodonCode Corporation, Massachusetts, USA). The assembled ITS rRNA gene sequence was queried against the NCBI nucleotide database using the Basic Local Alignment Search Tool (http://www.blast.ncbi.nlm.nih.gov/Blast.cgi). The isolate was identified as *M*. *mycetomatis* (GenBank: MW131313), showing 99% homology with a known isolate of *M*. *mycetomatis* (GenBank: MF980621).

The patient was referred to the dermatology ward for treatment. Antibacterial and antifungal therapy, respectively based on amoxicillin-clavulanic acid (1 g/125 mg tid) and terbinafine (500 mg bid), was started. However, the patient could only take the antibacterial treatment for 1 month due to limited financial resources. After 1 month and 15 days, he was discharged from the hospital. We were informed in a telephone follow-up call with his family that the patient had died 1 month after his discharge.

### Case discussion

Mycetoma continues to be endemic in the northern areas of West Africa including in Senegal. In this geographical area, it commonly affects the foot (70%) and the other most frequent extrapodal localisations are the leg, knee, thigh, hand, and arm (Box 1) [1]. Mycetoma is usually a localised subcutaneous mycosis which spreads locally along the fascial planes, thus affecting the skin, local deep structures, and bones. The spreading of the mycetoma agents via the local lymph nodes is rarely reported because few cases of blood-spread mycetoma have been reported (Box 1) [2,3]. Almost all of these rare cases of this form have been reported from Sudan, which can be considered as the most endemic country in the mycetoma belt [4]. Indeed, in 1972, Sudanese authors reported lymph node involvement in mycetoma representing 1.4% of a series of 949 mycetomas [5]. However, since the first report by Fahal and colleagues [6] in Sudan in 1996, disseminated eumycetoma from a subcutaneous mycetoma has continued to be reported by the Mycetoma Research Centre (MRC) at the University of Khartoum in Sudan. In 2016, multiple mycetoma lung secondaries to a knee eumycetoma, such as our present case, and mycetoma pulmonary secondaries to a gluteal eumycetoma were reported by teams from the MRC [2,3]. Similarly, in 2017, they reported broncho-pleuro-cutaneous fistula and pneumothorax as rare challenging complications of chest wall eumycetoma [7].

In Senegal, mycetoma is still endemic. Mycetoma mainly affects the foot, but the most frequent extrapodal localisations are the leg, knee, thigh, hand, and arm [1], although other unusual localisations are sporadically described, such as the scapulo-thoracic localisation reported by Sy and colleagues in 1998 [8], the orbital localisation reported by Gueye and colleagues in 2003 [9], and, more recently, a case of scalp mycetoma reported in 2019 (Box 1) [10]. Despite the fact that Senegal belongs to mycetoma belt, no cases of blood-spread eumycetoma have been reported in Senegal. However, 6 cases of lymph node involvement were evidenced by histology in a series of 109 cases between 1993 and 1998, all implicating actinomycetes agents [11]. To our knowledge, this is the first case of disseminated eumycetoma from a subcutaneous lesion to the lung in Senegal. Nevertheless, the limit of this case is that there is no evidence supporting the blood-spread eumycetoma. The length of the infection might explain the metastasis. Indeed, according to El Hassan and colleagues, lymphatic spread is less common in *M*. *mycetomatis* infection where the fungus is often enclosed in a fibrous capsule than in *Nocardia* and *Streptomyces* infections, where encapsulation is rare [5]. This assertion was confirmed by Develoux and colleagues, who stated that the most often implicated species in lymph node involvement in West Africa are *Actinomadura pelletieri* and *Streptomyces somaliensis*, due to the small size of their grains [1]. Again, this raise concerns around the delay in mycetoma management. As this case showed, patients lose a lot of time having their lesions treated by traditional practitioners before seeing a competent specialist who can diagnose the mycetoma. However, it must be noted that, even if a mycetoma is diagnosed at an early stage by a specialist, its management is still problematic in Senegal, due to the lack of competent health facilities (mycological diagnosis and systemic antifungal availability for treatment). Patients are often received at an advanced stage of the disease with surgical excisions or amputation as the only treatment options (Box 1) [4]. Unfortunately, our patient was no exception. The amputation was not unanimously accepted by all the orthopaedic staff, although it had been requested by the patient, because in sub-Saharan Africa, amputation is a social burden which can lead to death even excluding an infectious context.

Very often, the identification of mycetoma causing agents through culture leads to disappointing results. Several factors are incriminated such as insufficient quantities of grains for direct examination and culture, bacterial contamination, and unviable grains. In our case, the grains were sufficient, which corresponds to the normal situation for the identified species, *M*. *mycetomatis* [12]. However, culturing grains from sputum twice resulted in fungal contamination by *C*. *albicans*, despite a preliminary wash with physiological saline. The addition of a few drops of 10% povidone iodine was very useful for isolating the causative agent. Povidone iodine is an antiseptic and disinfectant, with broad-spectrum bactericidal, fungicidal, and virucidal action. It is bactericidal in less than 5 minutes in vitro on all bacteria and fungicide on yeasts (lethal on *Candida* in less than 1 minute in vitro) and filamentous fungi. Recommendations for washing grain in biological diagnosis of mycetoma suggest the addition of antibiotics for grains from eumycetoma [12]. However, in our case, even if we had used antibiotics, the result would be the same, as the contaminating agent was fungal. Thus, we intend to improve the process by testing the addition of povidone iodine at the same time to our standard procedure of washing the grains with physiological saline. The possibility of killing fungi using povidone iodine was previously recommended after debridement surgery to avoid the infection spread by Suleiman and colleagues [13].

Faced with the shortcomings of conventional methods for both the isolation and identification of mycetoma causative agents, laboratories in endemic countries are tending to adopt recent techniques as molecular tools (Box 1). We recommend that a cross sectional study among patients with eumycetoma should be conducted to determine the magnitude of such presentations. Depending on the results of such a study, the authorities should implement and fund a program aiming at facilitating access to antifungal molecules (Box 1).

**Box 1. Key Learning Points**

- Mycetoma is an NTD which is endemic in Senegal. Although this mycosis is most commonly found on the foot, extrapodal localisations have also been found, including on the leg, knee, thigh, hand, and arm.
- Spreading of mycetoma agents via local lymph nodes and resulting in bloodstream spread occurs, although it is rare.
- Patients with mycetoma should consult specialist in early stage of infection because in advanced stage of the disease, surgical excisions or amputation are the only treatment options.
- Due to the shortcomings of conventional methods for both the isolation and identification of mycetoma causative agents, laboratories in endemic countries should adopt recent techniques as molecular tools.
- The authorities should implement and fund a program aiming to facilitate access to antifungal molecules.
